# Global priorities for research and the relative importance of different research outcomes: an international Delphi survey of malaria research experts

**DOI:** 10.1186/s12936-016-1628-4

**Published:** 2016-12-06

**Authors:** Jo-Ann Mulligan, Lesong Conteh

**Affiliations:** 1 Research and Evidence Division, UK Department for International Development, 22 Whitehall, London, SW1A 2EG UK; 2 Health Economics Group, Department of Infectious Disease Epidemiology, School of Public Health, Faculty of Medicine (St Mary’s Campus), Imperial College London, Norfolk Place, London, W2 1PG UK

**Keywords:** Research assessment, Research priorities, Global health, Payback, Delphi, Malaria

## Abstract

**Background:**

As global research investment increases, attention inevitably turns to assessing and measuring the outcomes and impact from research programmes. Research can have many different outcomes such as producing advances in scientific knowledge, building research capacity and, ultimately, health and broader societal benefits. The aim of this study was to test the use of a Delphi methodology as a way of gathering views from malaria research experts on research priorities and eliciting relative valuations of the different types of health research impact.

**Methods:**

An international Delphi survey of 60 malaria research experts was used to understand views on research outcomes and priorities within malaria and across global health more widely.

**Results:**

The study demonstrated the application of the Delphi technique to eliciting views on malaria specific research priorities, wider global health research priorities and the values assigned to different types of research impact. In terms of the most important past research successes, the development of new anti-malarial drugs and insecticide-treated bed nets were rated as the most important. When asked about research priorities for future funding, respondents ranked tackling emerging drug and insecticide resistance the highest. With respect to research impact, the panel valued research that focuses on health and health sector benefits and informing policy and product development. Contributions to scientific knowledge, although highly valued, came lower down the ranking, suggesting that efforts to move research discoveries to health products and services are valued more highly than pure advances in scientific knowledge.

**Conclusions:**

Although the Delphi technique has been used to elicit views on research questions in global health this was the first time it has been used to assess how a group of research experts value or rank different types of research impact. The results suggest it is feasible to inject the views of a key stakeholder group into the research prioritization process and the Delphi approach is a useful tool for eliciting views on the value or importance of research impact. Future work will explore other methods for assessing and valuing research impact and test the feasibility of developing a composite tool for measuring research outcomes weighted by the values of different stakeholders.

**Electronic supplementary material:**

The online version of this article (doi:10.1186/s12936-016-1628-4) contains supplementary material, which is available to authorized users.

## Background

The last decade has seen increased attention to assessment and measurement of outcomes from research [1–6]. Research can have many different types of impact, including producing advances in scientific knowledge (through the production of papers and other published outputs, etc.), building research capacity, production of new products and devices, through to changes in policy, and ultimately health and societal gains. Funders of research are increasingly interested in methods for describing and quantifying these returns as well as gaining a better understanding of the different kinds of benefit or ‘payback’ from research outcomes [7]. As yet there is no agreed balance between the different dimensions of global health research output and no agreed generic measure of research performance that indicates whether one research project or programme has greater impact than another, or whether one set of research performance indicator values can be judged unequivocally to be an improvement on the results of previous years. Moreover, whilst there is a growing toolbox of methods for assessing and quantifying research impact, there is relatively little information on how different stakeholders value or prioritize the various outcomes of global health research. If development and research funding agencies wish to better understand how to maximize the outcomes of global health research, a first step is to better understand how different stakeholders value or rank the different outcomes from research.

The key to examining how people assess different research outcomes is to address the essentially subjective nature of the ‘trade-offs’ and to elicit how various stakeholders rank or prioritize outcomes from research. Stakeholders potentially include research funders, policy makers and the public, who are the ultimate ‘beneficiaries’ of research. Another important stakeholder group is the researchers themselves who not only ‘produce’ research, but through peer review, assess research outcomes, set research priorities and are themselves important users of the immediate outputs of research [8].

### Study objective

To understand views on research outcomes and priorities within malaria and across global health more widely, an international online Delphi survey of malaria research experts was conducted. The specific objectives of this study were to:

1. Contribute to the methodological literature on the assessment of research impact by applying the Delphi technique to the analysis of relative valuations of research outcomes;
2. Elicit rankings or values on different research outcomes;
3. Elicit rankings on research priorities within malaria and global health research.

The overall eventual aim (and the subject of future research) is to use the judgements on research outcomes to develop and test a methodological framework for assessing the impact of research investments of global health research.

## Methods

### The Delphi method

The Delphi process is a method by which input from individuals are elicited using a systematic, anonymous iterative approach [9]. The goal is to arrive at consensus around the topic in question.

The process works through a series of structured questionnaires (commonly referred to as ‘rounds’). The questionnaires are completed anonymously by ‘experts’ (commonly referred to as the panellists, participants or respondents). In the first round, the Delphi approach typically begins with an open-ended questionnaire to help set the parameters of the inquiry and identify which issues are of most importance to participants and researchers. In the second round each participant receives a second questionnaire and is asked to review the collated results from the first round. Participants are typically asked to rate or rank-order items to establish the relative importance among the different issues. As a result of round 2, areas of consensus and potential disagreement are identified. The Delphi process always involves at least two rounds but can extend to four or more rounds depending on the topic complexity. Importantly, the feedback process inherent in the Delphi technique allows and encourages the selected participants to re-assess their initial judgements about the information provided in previous iterations [10]. This gives the researchers more scope to follow up for clarification and further qualitative data [11]. Responses from panellists are always anonymous to everyone except the researcher. Finally, the Delphi group size does not depend on statistical power, but rather on group dynamics for arriving at consensus among experts. In this study the Delphi process was applied to invite expert opinions on the value of different types of research outcomes and research priorities in malaria and global health.

Delphi surveys have become a well-established technique for gathering expert opinions [12]. For instance, Fisk and colleagues used a Delphi exercise to assess absolute and relative government and charitable funding for maternal and peri-natal research in the UK and internationally [13]. In another study Fehr et al. employed an expert Delphi survey technique to examine and understand experts’ opinions on causes for lack of treatment options for neglected diseases and on feasible measures to promote research and development [14]. The survey also elicited expert opinions on the desirability of a regulatory instrument to promote research and development for neglected diseases. The Delphi approach has also been used to reach consensus on key parameters for public health and economic models relating to the outcome of untreated febrile illnesses [15].

### Study population

Panellists for the study were purposively selected based on their expertise and international reputation in malaria research, their involvement in the global malaria policy process and their relevant publications in the clinical and public health literature. The study aimed for a spread of experience from Europe, the USA, west, east, central and southern Africa and south and southeast Asia.

### Survey tool development

As a formative step, a half-day workshop was conducted with research leaders and experts in the field to develop broad questions to address global research priorities. This formed part of a wider public consultation process to assess research priorities [16]. To elicit views on the relative importance of different kinds of research outputs, a desk review of the various types of research impact and the models for classifying them was conducted. The review revealed a variety of theoretical frameworks and methodological approaches including, bibliometrics [2, 5, 6, 17]; so-called ‘payback’ and other multi-dimensional approaches [2, 4, 5]; economic approaches [1, 17]; and, case study approaches [2, 4, 6]. The payback framework was selected as the initial basis for eliciting views because it is multi-dimensional and very similar to the ways in which many research funders classify research output [18]. The payback framework is based around five categories of research impact: (a) knowledge production; (b) benefits for future research and research use; (c) informing policy and product development; (d) health sector benefits; and, (e) broader economic benefits (Box 1).

**Box 1:** The payback approach to classifying the different outcomes from research

Category descriptions
*Contribution to knowledge* e.g., the initial academic outputs from research such as journal articles; conference presentations; book chapters, and research reports
*Benefits to future research and research use* e.g., better targeting of future research; building research capacity in developing countries; other educational benefits
*Benefits from informing policy and product development* e.g., improved information bases for decision making; development of new drugs, vaccines, and other technologies
*Health and health sector benefits* e.g., improvements in health; improvements in the effectiveness and delivery of existing services
*Broader economic benefits* e.g., wider economic benefits from commercial exploitation of innovations arising from R&D; economic benefits from a healthy workforce

The framework was originally developed to examine the impact of UK health services research [18]. It was subsequently extended to examine basic and early clinical biomedical research [19, 20]. While the framework has been used to assess the impact of health research in the UK and North America, it has not been used widely to capture outcomes from global health or development research. Thus, a secondary aim of the study is to gain consensus on whether these are appropriate outcome categories or whether there are more appropriate categories of research outcome to consider.

### Survey process and content

The Delphi process followed the usual principles of anonymity, feedback and iteration [9]. The survey was conducted online in two rounds using *Qualtrics* online survey software [21]. Each expert received an individual email with a unique survey link to the questionnaire. This allowed follow-up with non-respondents. Respondents were sent two personalized reminders before the survey deadline. See Annex 1 for the full set of questions asked. Ethical approval for the study was obtained from the Imperial College Research Ethics Committee.

*Round 1* Invited experts were sent the round 1 questionnaire with an email cover letter in March 2015 and were given four weeks to complete the survey. The questionnaire began by asking respondents for their views on the most important malaria research developments in the last 20 years, then the most important malaria research priorities in the next 20 years and finally the top global health issues in the next 20–50 years.

To assess the relative importance of different types of research outcomes, the payback framework category descriptions were refined to ensure they were easy to interpret by the panel participants. Respondents were then asked to assign a percentage weight to each category, with the total summing to 100. This would force respondents to trade-off between the different categories. The questionnaire also invited respondents to make suggestions on whether to add or amend any of the existing categories.

Finally, panellists were asked to provide feedback on the survey structure and questions and basic demographic information, including location, area of work and years of relevant work or research experience were also collected. At the end of the first round, survey participants were informed that their results would be collated and fed back to them in a second round, where they would have the opportunity to revise their opinions if they so wished.

*Round 2* Experts that replied to the first round were sent a second questionnaire in June 2015 with three weeks to complete the survey. The questionnaire summarized the results from the first round and participants were invited to modify their responses in the light of the anonymized group responses. The format for the second round differed from the first round in that panellists were asked to indicate how important they considered each of the developments identified in the first round on a five-point ‘Likert’ scale: from ‘most important’, ‘important’, ‘less important’, ‘least important’, to ‘no judgement’. Participants were able to rank more than one issue in the top ten issues presented as ‘most important’. The use of the Likert scale as opposed to free text responses was chosen in the second round because the responses are easily quantifiable and the technique is readily understandable by participants [22]. Another advantage of Likert scales over free text responses is they enable respondents to respond in degrees of agreement (which makes answering questions easier) as well as to allow for undecided or neutral feelings about the topic of interest [23]. The introduction letter to participants and survey tools are provided in Additional File 1.

## Results

### Participation, attrition and demographic data

**Fig. 1:**
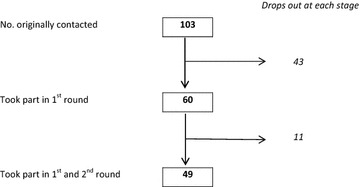
Respondents/non-respondents at each stage of the process

**Fig. 2:**
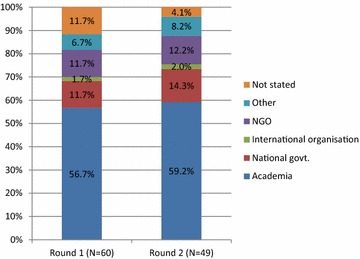
Professional affiliation of participants

**Fig. 3:**
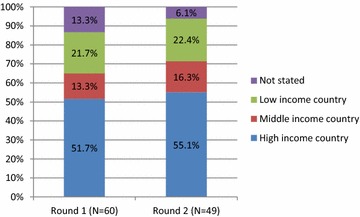
Place of residence of participants

**Fig. 4:**
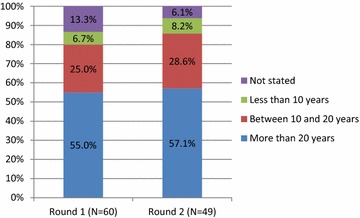
Years of relevant work experience of participants

Of the 103 malaria research experts originally contacted, 60 (58%) initially agreed to participate and completed the first questionnaire, and 49 experts (48.5%) completed both the first and second round questionnaire (Fig. 1). Professional affiliations of malaria research experts in both rounds were similar with the majority of participants working in an academic environment (Round 1: 56.7%; Round 2: 59.2%) (Fig. 2). Residency of participants was also similar in both rounds with most participants indicating their place of residence in a high-income country (Round 1: 51.7%; Round 2: 55.1%) (Fig. 3). Most participants had more than 20 years’ relevant work experience in both rounds (Round 1: 55.0%; Round 2: 57.1%) (Fig. 4).

**Fig. 5:**
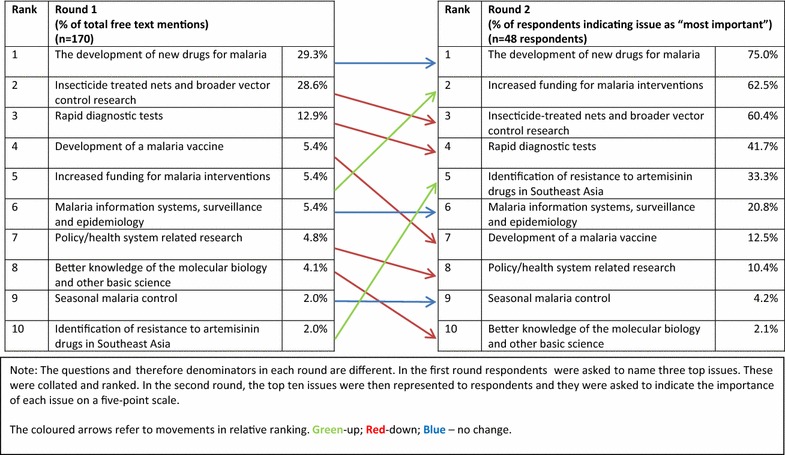
Ranked most important past developments in malaria research

The first question asked for views on the most important research developments in the last 20 years. In the first round, these were free text responses and participants were invited to list up to three separate developments. The responses were then coded and categorized. In the second round, the top ten research developments from the first round were presented back to experts in order of importance (i.e., percentage mentioning as a top-three issue). Experts were then asked to indicate how important they considered each development to be on a five-point scale: ‘most important’, ‘important’, ‘less important’, ‘least important’, and ‘no judgement’ (Fig. 5).

The development of new drugs for malaria and insecticide-treated nets were the top two most important developments in malaria research in both the first and second round. Although the question was focused on research developments, many experts in the first round cited the importance of increased funding for malaria control more generally as an important influence on research. This was confirmed in the second round with 29 (60.4%) experts mentioning it as ‘most important’. The survey also asked respondents (in the first round only) to tell us what ‘surprised’ them most about developments in malaria research in the last 20 years. These were free text responses to add qualitative context to the earlier question on what they considered to be the most important past developments within malaria research. This elicited a wide range of responses covering many different themes. The theme most frequently mentioned by respondents was the failure to develop an effective malaria vaccine with ten out of the 60 respondents referencing it some way. Other themes included the speed in progress in reducing malaria mortality from the deployment of existing tools (eight respondents) and the lack of investment and innovation in vector control research (six respondents). Box 2 shows a selection of comments that capture the variety of free text responses. The full range of responses are provided in Additional file 2.

**Box 2:** A selection of free text responses to the question: “What has surprised you most about developments in malaria research in the last 20 years?”

“The limited progress made in developing a truly effective malaria vaccine”. (other professional affiliation, high-income country)
“The continued investments in vaccines and genomic solutions at the expenses of investments in vector control which have saved more children (than) any other intervention in the present and the previous malaria elimination campaigns” (Academia, high-income country)
“That operational research is not more important” (Academia, high-income country)
“Low interest and lack of funding in malaria compared to new diseases such as HIV, few innovations in new effective malaria control tools” (National government, low-income country)
“The unprecedented rise in the investment in malaria research in malaria endemic countries largely due to support from external funders. Despite this global effort how relatively little domestic funding government in malaria endemic countries continue to invest in malaria research” (Academia, low-income country)
“After working for 30 years on malaria control in Africa I was amazed when malaria started to decline in many African countries” (Academia, high-income country)
“Most surprising has been that lack of “true innovation” in malaria community. Our current best tools are still also our oldest” (Non-government organization, low-income country)
“The enormous disparity between research and policy, and the parallel universes of the research and international health/donor communities” (Academia, low-income country)
“The little investments in health systems research—understanding how health systems could improve effective coverage of malaria interventions” (Academia, high-income country)
“The slow progress from research into policy following the discovery of ACT while there was evidence that chloroquine resistance was associated with increased mortality” (Academia, middle-income country)

### Views on priority areas for malaria research investment in the next 20 years

**Fig. 6:**
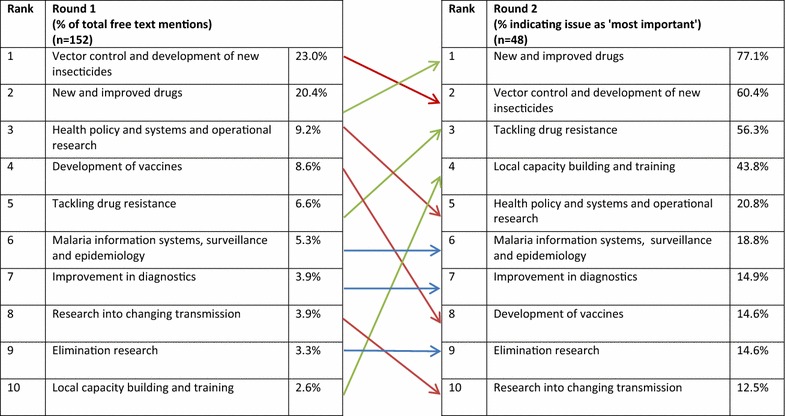
Ranked priority areas for malaria research investment in the next 20 years

The next question asked experts to identify the priority areas for malaria research investment in the next two decades. In the first round experts were invited to suggest up to three areas for priority investment. The results were collated and the top ten suggestions were presented back to participants in the second round as a ranked list of possible research investments. In the second round new and improved drugs was identified by 77.1% of the panel as the most important area for future research investment followed by vector control and the development of new insecticides (60.4% citing it as most important) (Fig. 6). Tackling drug resistance was ranked the third most important issue with 56.3% of participants citing it as ‘most important’. Although investment in research capacity building was only the tenth most frequently mentioned issue in the first round, it was promoted to the fourth most important area for investment in the second round.

### Views on global health issues in the next 20–50 years

**Fig. 7:**
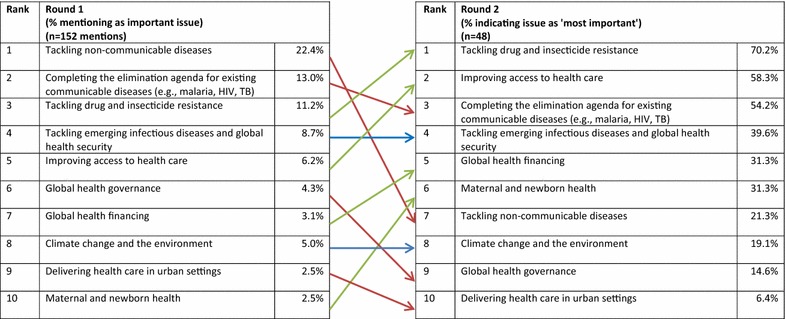
Ranked most important global health issues in the next 20–50 years

This next section asked experts to consider wider issues in global health in a longer timeframe. Here, there were notable differences between responses in rounds 1 and 2. The first round asked experts to identify the three most important issues for global health in the next 20–50 years. Non-communicable diseases (NCDs) in its various forms was the most frequently mentioned issue in the first round but dropped to seventh place when presented back to participants in the second round (Fig. 7). Tackling drug and insecticide resistance emerged as the biggest issue in the second round with 70.2% of experts citing it as ‘most important’ followed by improving access to health (58.3%) and completing the elimination agenda for existing communicable diseases (54.2%). Participants (in the first round only) were also asked what issue of global health importance today would be *less* important in the future. The top three issues were dominated by communicable diseases (Table 1).

**Table 1:** In your opinion, what issues of global health importance today will be *less important* in the future?

Description	No. of mentions (n = 44)	%
Communicable diseases and the ‘classical infections’	16	36
Neglected tropical diseases	5	11
HIV management	4	9
Diseases related to hygiene and sanitation, such as cholera	2	5
New emerging diseases	2	5
Inequities	1	2
Vaccines aimed at reducing mortality rather than interrupting or eliminating transmission	1	2
Car accidents	1	2
Famines	1	2
Fundamental studies of immunology, vaccinology and transgenics	1	2
Cancer and obesity	1	2
Commodity procurement	1	2
Don’t know	8	18

Question asked in round 1 only

### Views on the importance of different research outcomes

The last question addressed expert views on the relative value or importance of different types of research outcomes and provided an opportunity to critique the five pre-determined payback framework categories. The categories ‘informing policy and product development’ and ‘health and health sector benefit’ were weighted the highest in the first round with very similar mean values (25.4 and 25.6%, respectively) (Table 2). ‘Broader economic benefits’ were considered the least important category (12.9%). In the second round, the first round results (rounded to the nearest whole number) were presented to experts and asked whether they broadly agreed with the average weightings from the panel or whether they wished to adjust the results. Respondents were also asked whether they were broadly content with the payback framework as a system for classifying research outcomes.

**Table 2:** What *percentage weight* would you give to each of the following outcome categories, in terms of their importance to overall research impact?

Research outcome category	Round 1 (n = 54)		Round 2 (n = 49)	
	Mean value	Std. err.	Mean value	Std. err.
Contribution to knowledge	19.6	2.47	18.9	0.53
Benefits to future research and research use	17.3	1.06	16.6	0.43
Benefits from informing policy and product development	25.4	1.56	25.4	0.26
Health and health sector benefits	25.6	1.45	26.3	0.40
Broader economic benefits	12.0	1.04	12.9	0.42

The results in the second round were similar to those in the first, with the majority of respondents (78%) agreeing with the average weights presented from the earlier round. Those that disagreed were invited to submit revised weights. The impact of the revised weights was to slightly decrease the weight given to ‘contribution to knowledge’ and ‘benefits to future research’ and increase the weight to ‘health and health sector benefits’ and ‘broader economic benefits’ (see Table 2). However ‘informing policy and product development’ and ‘health and health sector benefits’ remained the highest weighted research outcomes in round 2 with very similar mean values to those reported in round 1 (25.4 and 26.3%).

Analysis by background and location offered some insights as to how different groups might weight different research outcomes, although a much bigger sample size would be needed to reveal significant differences. Those working in academic institutions on average gave a higher weight to ‘contribution to knowledge’ than those working in government or NGOs (see Additional file 3). However, all professional groups gave their highest average weighting to ‘health and health sector benefits’. Those working in high and middle income settings were more likely to give the highest weight ‘to informing policy and product development’ while those working in lower income settings weighted ‘health and health sector benefits’ the highest. In terms of number of years of relevant experience (i.e., fewer than ten years; between ten and 20 years; and more than 20 years), all three groups weighted ‘health and health sector benefits’ the highest. That said, it is important to reiterate that the purpose of the Delphi was not to provide a quantitative analysis of these differences, but rather to gain the group’s *overall* judgement on the relative weight of each research outcome category.

Most respondents (66.6%) were content with the way in which research outcomes were classified according to the payback framework. Of those that provided suggestions for refinement, several suggested categories that were already captured in the framework, such as benefits to research capacity building (six respondents) or economic benefits (two respondents). Suggestions for new research outcome categories not already captured by the payback framework, included ‘environment benefits’ (two respondents), ‘benefits for sustainability’ (one respondent) and ‘elevating awareness of public health problems’ (one respondent). However, as the numbers suggesting changes were small, the research outcome classification system was left unchanged for the second round.

## Discussion

### Methods

On the basis of the response rate and comments, the Delphi method and the online survey approach were generally well received. Experts were willing to devote time and effort to complete two online surveys over five months and also provided helpful comments. Several researchers commented that it was good to have the opportunity to participate in a transparent approach to priority setting. The literature recommends anything from 10 to 80 experts on a Delphi panel [9, 11] and the study response rates compare favourably with other recent online Delphi surveys. For instance, an expert online Delphi survey on research and development into drugs for neglected diseases reported first and second round response rates of 30% (117/388) and 14% (56/388), respectively [14]. The sample selection was purposive in that it identified interested experts from specific sources, such as global malaria initiatives, review committees, conference presentations, and relevant publications. There was no notable difference in demographic characteristics of participants between the two rounds. Both the first and second round samples were biased towards those working in academia and more than half stated their place of residence in a high-income country. As with other Delphi studies, it is impossible to judge how and if the results may have differed if the panel characteristics were different [14]. There was some attrition in the response rates over the two rounds, with 11 participants dropping out between the first and second survey. However, as the characteristics of those completing both surveys were broadly similar it is unlikely that drops-outs overly influenced the nature of responses.

There are several strengths in the Delphi method as a structured process for gathering opinions. First, it enables input from individuals using a systematic anonymous and iterative approach to gain consensus on an area of interest. This is likely to allow a more inclusive process of determining values than focus group discussions [10]. Delphi surveys also allows for a range of individuals to express their opinion which can then be reassessed by considering the input from other participants, with the eventual aim of reaching some convergence [15]. A significant advantage of the Delphi is that participants are very much aware at each stage of the results of the previous survey rounds, and there is scope for each panel member to provide more detailed feedback on both the process and the results.

While the Delphi method has several appealing characteristics, there are limitations. First, the approach, as noted earlier, is not designed to be statistically representative. Therefore, it is not possible to generalize these findings to the population of malaria research experts or researchers in general. Other more quantitative techniques, such as discrete choice experiments, offer a way to systematically assess how groups value different types of research outcomes, although they are time consuming, require a lot of data and can be expensive to undertake. A second drawback of the Delphi approach is it can be a lengthy process due to its iterative nature. Linked to that, the multiple feedback processes, integral to the Delphi concept, mean the problem of low response rates are magnified. If a certain group of experts discontinue their responses during various stages of the Delphi process, the quality and reliability of the information obtained could be threatened [10]. A potentially more serious issue is that the iteration characteristics of the Delphi technique can potentially enable investigators to ‘mould’ opinions. One experiment conducted in the 1970s by Scheibe and colleagues showed that Delphi subjects would rate their responses differently after receiving distorted feedback [24]. So-called ‘acquiescence’ bias is a particular risk when asking respondents whether they agree with the presented findings from the first round.

A further drawback is an assumption that Delphi participants “are equivalent in knowledge and experience” [25]. There are good reasons for concern that the knowledge of participants could be unevenly distributed, whereby some participants have much more expertise in certain areas than others. Experts who have less in-depth knowledge of certain topics may be unable to distinguish the most important statements that have been identified by those subjects who possess wider knowledge. In these instances the outcomes of a Delphi study could be the result of identifying a series of ‘general’ issues rather than an in-depth exploration of the topic. While this is certainly a risk, it is arguably a risk for all qualitative investigations that seek to gather some kind of group consensus. None of the issues or questions raised by the Delphi were thought to beyond the expertise of any of the participating experts.

Another potential criticism of the study approach is that the final sample was biased towards research experts largely working in academic institutions. It is acknowledged that malaria research, particularly operational research, is also conducted outside of traditional academic settings, such as in non-governmental organizations or other implementing agencies. It could be the case that a sample with a more diverse background could have led to different results, such as an even higher weighting on later stage impact. Future work could explore the views of those working in other settings.

The dominance by northern academics based in high-income countries means there may be a bias in the round 1 free text mentions that was propagated to round 2, given the structure of the iterative process. On reflection more information on the areas of particular interest of panel members could have been requested. However, it might have been hard for experts to narrowly define their interests. It is also likely that many panel members had overlapping interests, making interpretation difficult.

### Research priorities

The study highlighted the importance of past research developments in malaria, particularly the development of new drugs, insecticide-treated bed nets and broader vector control. However, experts were also keen to highlight that increased funding for malaria interventions more generally were critical to improvements in the scientific knowledge base. Priorities for future malaria research investment reflected areas where the most success had been seen in the past (vector control and new drugs), but also highlighted the importance of tackling emerging drug and insecticide resistance, in particular resistance to artemisinin-based combination therapy. Experts agreed that staying ahead of the challenge of resistance requires expanding the pipeline of new drugs, diagnostics and other tools. One participant commented that there has been a lack of “true innovation” in the community, arguing that “our current best tools are still also our oldest”. Local capacity building and wider health systems and operational research were also highlighted. This possibly reflects a more general concern that there is a potential ‘disconnect’ between researchers and implementers and there are insufficient people with the skills to bridge this gap. One expert commented that funders are more willing to fund the clinical science with less supporting operational or implementation research.

Looking beyond malaria, experts identified tackling broader drug resistance (i.e., antimicrobial and antibacterial) as the most important area for attention in the next 20–50 years. This reflects wider political and global concern on the growing threat of resistance; both the UK and WHO have raised this issue as a global threat [26, 27]. The one case where there was apparently less congruence between the two rounds was on the importance of NCDs. NCDs was the most frequently mentioned future global issue in the first round, but participants ranked NCDs lower (seventh) in the second round when it was presented alongside other issues. Most obviously, this may reflect the disciplinary bias of the group, who all worked in infectious disease. But there is little doubt that chronic NCDs are reaching epidemic proportions worldwide [28]. NCDs currently make up more than 40% of the disease burden and are expected to overtake communicable diseases as the major cause of mortality in low-income countries (LICs) by 2030 [29]. Indeed, the emergence of NCDs in LICs is partially the result of progress in tackling high-burden communicable diseases such as HIV/AIDs, tuberculosis and malaria with the resultant increase in average life expectancy in many countries [30]. However (to participants at least) it may be that the perception of the NCD burden in sub-Saharan Africa is still overshadowed by the unfinished agenda on communicable diseases as well as tackling emerging infectious diseases of epidemic potential, such as Ebola.

### Valuing research outcomes

A key aim of the Delphi survey was to elicit expert views on the relative importance of different global health research outcomes. In this context ‘value’ is equated to percentage weight assigned to each outcome category. The findings suggest that malaria research experts prioritized research outcomes that focused most on ‘health and health sector benefits’ and ‘informing policy and product development’. The overall rankings did not change over the two rounds of the survey, although there were some differences within each round when the results were broken down by experience, region and employment sector. ‘Contribution to scientific knowledge’ came lower down the priority list, suggesting that participating experts valued efforts to move research discoveries to health products and services over pure advances in scientific knowledge. Broader economic outcomes were valued the least important. The finding that economic outcomes are weighted less highly than health outcomes is perhaps not that surprising. This is a similar finding to a previous study looking at the outcomes of medical research in Canada [8]. The authors suggest this could reflect researcher views on the limited potential of research to generate pure economic returns, but it could also reflect the lack of data on the economic returns of health research more generally, malaria research in particular. Similarly, a more recent study conducted in the UK suggested that: “achieving higher life expectancy for adults living with a common chronic disease in the UK is one of the highest priorities for the general public and researchers—well ahead of commercial and employment benefits (from the research)” [31]. That said, the same study found that researchers and the general public agreed that creating substantial numbers of jobs through research is important, suggesting that perceptions of economic impact can be more nuanced. Exploring the economic rates of return from global health research will be the subject of future work.

It is worth keeping in mind that the different outcomes from research are not mutually exclusive, and are often interlinked in a logic chain from a research award or grant, through to research outputs through to impact. A research programme that performs well on one dimension, for example building research capacity, may mean performing less well on another dimension, such as publishing high-impact journal articles. Nonetheless, the findings suggest that more value should be placed on later stage impact indicators with participants giving a combined weight of 36% to the two indicators (i.e., health and health sector benefits + broader economic benefits). This is higher than the impact weight of 20% assigned by the UK Research Excellence Framework [32]. A key question, inevitably, is how to accurately identify, attribute and measure those impacts.

## Conclusions

The Delphi process was found to be a reasonably straightforward approach for eliciting views on research priorities, particularly when compared to more intensive priority setting exercises, such as the Malaria Eradication Research Agenda (malERA), which over the course of two years involved more than 250 experts from 36 countries in 20 face-to-face meetings around the world [33]. While the approach certainly has limitations, it could be a useful low-cost complement to these large-scale endeavours, particularly if undertaken on sub-topics within malaria.

The assessment of the wider impact of research is becoming a firmly established field. The UK in many ways has been at the forefront of efforts to take a more systematic approach to assessing impact by asking researchers to include four-page impact case studies in their submissions to the 2014 Research Excellence Framework [34]. During this process, the research of 154 English higher education institutes was assessed and graded, with the results informing the allocation of around £1.6 billion of research funding [31]. This study shows that it is possible to arrive at a degree of consensus on the weight or value of different types of research outcome using the Delphi technique amongst a particular stakeholder group (malaria research experts). While there have been previous studies examining the relative valuations of different kinds of research impact [8, 31], this is the first study to consider global health research relevant to people living in developing countries. Future work will explore the extent to which it is possible to develop a composite research performance indicator, by applying the weights derived in the study to a sample of research outputs to see whether it changes the rankings for the valuation of research more generally. However, a key issue is the extent to which different stakeholders might value different research outcomes in different ways. As Pollitt et al. also note, this is vital to know as funders are actively encouraging research groups to take into account the wider benefits of research [31]. More work is needed to validate and build upon these and previous findings, particularly for global health research. One possibility is to repeat the exercise with different stakeholder groups and/or consider the use of more quantitative techniques, such as discrete choice experiments, to derive more robust comparative estimates of relative value across a broader range of research beneficiaries. Nonetheless it is hoped the findings presented here will add to the global evidence base on how stakeholder views could, or should, influence judgements on the overall performance of publically funded global health research.

## Additional files

**Additional file 1.** Introduction letter and survey tools.

**Additional file 2.** Surprising developments in malaria research: free text responses.

**Additional file 3.** Mean research outcome weights by background.

## Abbreviations

DFID: Department for International Development
HIV/AIDS: human immunodeficiency virus/acquired immune deficiency syndrome
LICs: lower income countries
NCDs: non-communicable diseases
NGO: Non-Government Organisation
R&D: Research and Development
R1: round 1
R2: round 2
WHO: World Health Organization
