# Supplementary material for: Global priorities for research and the relative importance of different research outcomes: an international Delphi survey of malaria research experts
Source: Malar J. 2016 Dec 6;15:585. doi: 10.1186/s12936-016-1628-4 (PMC5139033; doi:10.1186/s12936-016-1628-4)
Supplement: Supplementary file 1 — Additional file 1. Introduction letter and survey tools. [file 12936_2016_1628_MOESM1_ESM.docx]

**Additional File 1: Introduction letter and survey tools**

**Delphi introduction letter to participants**

Dear Colleague
 
We have identified you as a leading expert in malaria research, and seek your assistance in participating in a 'Delphi' exercise on the impact of malaria research and what should be our research priorities in the next 5 – 20 years. The findings from the survey will be important in helping the UK Department for International Development (DFID) design and commission research in the future.
 
Delphi surveys are a structured group interaction process that involves two or more 'rounds' of opinion collection and feedback.  For this Delphi survey, we are using a short online questionnaire developed in collaboration with Imperial College London. It should take no more than 10 minutes to complete.
 
Follow this link to the survey:
${l://SurveyLink?d=Take the Survey}

Or copy and paste the URL below into your internet browser:
${l://SurveyURL}

We would be grateful if you can complete the survey by **Friday 27th March 2015**.  Once we have analysed the results we will feedback your anonymous responses in relation to those of other experts, for you to modify your answers should you wish, before sharing the final results.
 
The knowledge gained from this survey will be shared in summary form, without revealing individuals’ identities. With your agreement, anonymous quotes may be used to illustrate general points, but if they are used they will not contain any information that will allow identification of individuals.
 
By clicking on the link above you consent to take part in the survey and to the summary results being published.
 
We do hope you agree to participate in this survey.  If you have any questions please do not hesitate to contact me at [j-mulligan@dfid.gov.uk](mailto:j-mulligan@dfid.gov.uk)
 
Yours sincerely

Jo Mulligan
 
Senior Health Adviser
Human Development Team
Research and Evidence Division
UK Department for International Development

Follow the link to opt out of future emails:
${l://OptOutLink?d=Click here to unsubscribe}


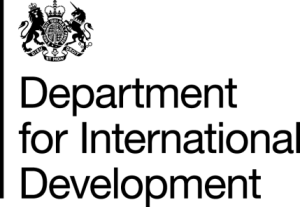


**First round survey questionnaire**

Q1 Welcome and thank you for supporting this Delphi survey on malaria research priorities and outcomes.

Delphi surveys allow a range of individuals to express their opinion which can then be reassessed by considering the input from other participants, with the eventual aim of reaching some convergence.   The survey should take about 10 minutes to complete.

Please click on >> below to start the survey

Q2 Thinking first about past progress in malaria, what do you consider to be the most important developments in malaria research in the last 20 years? These can be methodological, empirical, technological or policy related. You may list up to three.

Research development 1 (1)

Research development 2 (2)

Research development 3 (3)

Q3 What has surprised you most about developments in malaria research in the last 20 years?

*[free text]*

Q4 What should be the priority areas for malaria research investment in the next 20 years? You may list up to three.

Research priority 1 (1)

Research priority 2 (2)

Research priority 3 (3)

Q5 Thinking about global health more broadly, what do you think will be the top three global health issues in the next 20 years?

Global Health Priority 1 (1)

Global Health Priority 2 (2)

Global Health Priority 3 (3)

Q6 In your opinion, what issues of global health importance today will be less important in the future?

*[free text]*

Q7 We now want you to consider the different outcomes from research investments.   Global health research can lead to a variety of benefits. One way of thinking about these benefits is to use the ‘Payback Framework’ which divides research into five different outcome categories.    What percentage weight would you give to each of the following categories, in terms of their relative importance to the overall impact of a research programme?

(Note: your values must total 100).

- ______ Contribution to knowledge: e.g. the initial academic outputs from research such as journal articles; conference presentations; book chapters and research reports.   (1)
- ______ Benefits to future research and research use: e.g. better targeting of future research; building research capacity in developing countries; other educational benefits.   (2)
- ______ Benefits from informing policy and product development: e.g. improved information bases for decision making; development of new drugs, vaccines, and other technologies.   (3)
- ______ Health and health sector benefits: e.g. improvements in health; improvements in the effectiveness and delivery of existing services.   (4)
- ______ Broader economic benefits: e.g. wider economic benefits from commercial exploitation of innovations arising from R&D; economic benefits from a healthy workforce.   (5)

Q8 In addition to those categories previously listed, are there any categories you would like to add or amend? If so, please describe below.  (if necessary use the back button << to review)

Q9 You have completed the main part of the questionnaire. We now want to ask a few final questions about the survey process and your background.   Do you have any comments on this Delphi survey or on the questionnaire?

Q10 What is your current professional affiliation?

- Academia (1)
- National government (2)
- Industry (3)
- International organisation (4)
- Non-governmental organisation (5)
- Other (6) ____________________

Q11 Your place of residence is in a

- High income country (1)
- Middle income country (2)
- Low income country (3)

Q12 How many years of relevant work or research experience do you have?

- Less than 10 years (1)
- Between 10 and 20 years (2)
- More than 20 years (3)

Q13 Thank you for participating in the first round of this Delphi survey. We will now evaluate the outcome of this round.   All participants who completed the first round will receive an email containing the link to the questionnaire for the second round of this survey.   In the second round, you will see the results of this first round as well as comments and suggestions which the participating experts have added to this questionnaire.   Please click on the >> button below to save your responses.

**Second round survey questionnaire**

Q1 DFID Survey on Research Priorities and Outcomes Welcome and thank you for supporting this second round of our Delphi survey.   We have just five questions to ask you. In this round we will show you the overall results from the first round and invite you to review and provide further input, with the eventual aim of reaching some convergence.

Please click on >> below to start the survey

Q2 Past developments in malaria research

We asked you to identify the most important developments in malaria research in the last 20 years. Based on responses from the survey, the top 10 research developments are listed below in rank order. Please indicate how important you think each development has been.

|  | Most important (1) | Important (2) | Less important (3) | Least important (4) | No judgement (5) |
| --- | --- | --- | --- | --- | --- |
| 1. The development of new drugs for malaria (1) |  |  |  |  |  |
| 2. Insecticide treated nets and broader vector control research (2) |  |  |  |  |  |
| 3. Rapid diagnostic tests (3) |  |  |  |  |  |
| 4. Development of a malaria vaccine (4) |  |  |  |  |  |
| 5. Increased funding for malaria interventions (5) |  |  |  |  |  |
| 6. Malaria information system, surveillance, epidemiology (6) |  |  |  |  |  |
| 7. Policy/health system related research (7) |  |  |  |  |  |
| 8. Better knowledge of the molecular biology and other basic science (8) |  |  |  |  |  |
| 9. Seasonal malaria control (10) |  |  |  |  |  |
| 10. Identification of resistance to artemisinin drugs in South East Asia (11) |  |  |  |  |  |

Q3 Optional: If you have any further comments on this question, please add here

*[free text]*

Q4. Future priorities for malaria research

We asked you what should be the priority areas for malaria research investment in the next 20 years. Based on responses from the survey, the top 10 priorities are listed below in rank order.  Please indicate how important you think each priority is.

|  | Most important (1) | Important (2) | Less important (3) | Least important (4) | No judgement (5) |
| --- | --- | --- | --- | --- | --- |
| 1. Vector control, development of new insecticides and research on insecticide resistance (1) |  |  |  |  |  |
| 2. New and improved drugs (2) |  |  |  |  |  |
| 3. Health policy and systems and operational research (3) |  |  |  |  |  |
| 4. Development of vaccines (4) |  |  |  |  |  |
| 5. Tackling drug resistance (5) |  |  |  |  |  |
| 6. Malaria information system, surveillance, epidemiology (6) |  |  |  |  |  |
| 7. Improvement in diagnostics (7) |  |  |  |  |  |
| 8. Research into changing transmission (8) |  |  |  |  |  |
| 9. Elimination research (10) |  |  |  |  |  |
| 10. Local capacity building and training (11) |  |  |  |  |  |

Q5 Optional: If you have any further comments on this question, please add here

*[Free text]*

Q6 Future global health issues.

We asked you to identify what are likely to be the most important global health issues in 20-50 years time.  Based on responses from the survey, the top 10 global health issues are listed below in rank order. Please indicate how important you think each issue is.

|  | Most important (1) | Important (2) | Less important (3) | Least important (4) | No judgement (5) |
| --- | --- | --- | --- | --- | --- |
| 1. Tackling non communicable diseases (1) |  |  |  |  |  |
| 2. Completing the elimination agenda for existing communicable diseases (e.g. malaria, HIV, TB) (2) |  |  |  |  |  |
| 3. Tackling drug and insecticide resistance (3) |  |  |  |  |  |
| 4. Tackling emerging infectious diseases and global health security (4) |  |  |  |  |  |
| 5. Improving access to health care (5) |  |  |  |  |  |
| 6. Global health governance (6) |  |  |  |  |  |
| 7. Global health financing (7) |  |  |  |  |  |
| 8. Climate change and the environment (8) |  |  |  |  |  |
| 9. Delivering health care in urban settings (11) |  |  |  |  |  |
| 10. Maternal and newborn health (12) |  |  |  |  |  |

Q7 Optional: if you have any further comments on this question, please add here

[Free text]

Q8 Relative valuation of research outcomes

We asked you to think about the different outputs from a typical research programme and to tell us what percentage weight you would give to each category, in terms of their importance to overall research impact.

The average values from participants are provided below.  Percentage weight (mean value from respondents)

- Contribution to knowledge (e.g. the initial academic outputs from research such as journal articles; conference presentations; book chapters and research reports)  20%
- Benefits to future research and research use (e.g. better targeting of future research; building research capacity in developing countries; other educational benefits) 17%
- Benefits from informing policy and product development (e.g. improved information bases for decision making; development of new drugs, vaccines, and other technologies) 25% Health and health sector benefits (e.g. improvements in health; improvements in the effectiveness and delivery of existing services) 26%
- Broader economic benefits (e.g. wider economic benefits from commercial exploitation of innovations arising from R&D; economic benefits from a healthy workforce)  12%

Do you broadly agree with these percentage weights?

- Yes (1)
- No, I would like to adjust these values (2)

*[For those that answered Yes]*

Q9 Please tell us what percentage weight you would give to each of the following outcome categories, in terms of their importance to overall research impact. The previous answers from the survey panel are in brackets.

Note your answers must sum to 100.

- Contribution to knowledge: e.g. the initial academic outputs from research such as journal articles; conference presentations; book chapters and research reports (Panel response: 20%).   __________
- Benefits to future research and research use: e.g. better targeting of future research; building research capacity in developing countries; other educational benefits (Panel response 17%).   _________
- Benefits from informing policy and product development: e.g. improved information bases for decision making; development of new drugs, vaccines, and other technologies (Panel response: 25%).   __________
- Health and health sector benefits: e.g. improvements in health; improvements in the effectiveness and delivery of existing services (Panel response: 26%). ________
- Broader economic benefits: e.g. wider economic benefits from commercial exploitation of innovations arising from R&D; economic benefits from a healthy workforce (Panel response 12%).   __________

Q10. You have almost finished the survey, if you have any further comments on any aspect, please add here:

*[Free text]*

Q11. You have finished the survey! We will now evaluate the outcome of this 2nd round and share the results with you in due course.

Please click on the >> button below to save and send your responses
