# Supplementary material for: Global priorities for research and the relative importance of different research outcomes: an international Delphi survey of malaria research experts
Source: Malar J. 2016 Dec 6;15:585. doi: 10.1186/s12936-016-1628-4 (PMC5139033; doi:10.1186/s12936-016-1628-4)
Supplement: Supplementary file 2 — Additional file 2. Surprising developments in malaria research: free text responses. [file 12936_2016_1628_MOESM2_ESM.docx]

**Additional File 2: All responses to the question: “What has surprised you most about developments in malaria research in the last 20 years?”**

**Responses from Academia**

| “The realisation that Plasmodium vivax is actually a very dangerous infection that is probably responsible for a great deal more morbidity and mortality than we as a research community ever anticipated.” |
| --- |
| “Two things: / 1. That despite all the progress, the same old targets are still there as vaccines and drugs, being championed by the same big shots (naming no names). And this is in spite of evidence that several of these (vaccines in particular) have been shown to be failures. / 2. That despite the massive developments and innovations in technologies there is still so much weak science on the malaria parasite out there. This should not be acceptable just because researchers are working on what has previously been perceived as a "hard" or "genetically intractable" parasite.” |
| “from control to elimination” |
| “The continued investments in vaccines and genomic solutions at the expenses of investments in vector control which have saved more children that any other intervention in the present and the previous malaria elimination campaigns” |
| “the speed in shifting the research focus to malaria elimination agenda” |
| “Understanding of the molecular mechanisms related to the pathogenesis of infection, e.g. placenta malaria.” |
| “The large focus on immunology and vaccine development, which despite the huge efforts and huge resources invested has not delivered an effective vaccine or other useful intervention” |
| “No novel chemical insecticides in the last 20 years” |
| “that operational research is not more important” |
| “The renewed interest in malaria elimination, something not even considered 10 yrs ago.” |
| “There have been no major surprises. Progress in the development of new antimalarials, insecticides and vaccines has been slow but it is not surprising that this has been the case. It might have been anticipated that more direct benefits would have emerged from the genome projects than has so far been the case.” |
| “Development of capacity to conduct good clinical trials in the field of malaria in many sites in Africa and in Asia. Yet several countries/geographical areas lack basic infrastructure to conduct malaria research. We have rather expanded existing structure” |
| “The number of questions that still remain to be answered - and the lack of historical perspective of many of those coming new to the discipline.” |
| “Lack of support by international development agencies especially DfID for malaria vaccine development.” |
| “Rapid acceptance of new tools and concepts, sometimes over remarkably short time spans” |
| “1. Large scale funding for mass roll-out of vector control. / 2. Development and large scale use of rapid diagnostic tests. / 3. Reluctance to accept that IRS is a highly effective method of malaria prevention, AND that IRS provides added protection in addition to use of LLINs. / 4. The slow development of new paradigms of vector control on the hand, and the poor quality of many entomological trials that attempt to show effectiveness of new tools. /” |
| “How definitions of artemisinin resistance that are not wholly rigorous can dominate scientific and funding agendas” |
| “Ability to reduce mortality. Failure of vaccines. Still limited therapeutic options” |
| “the number of (young) researchers coming into the field. A very good thing!” |
| “After working for 30 years on malaria control in Africa I was amazed when malaria started to decline in many African countries.” |
| “1. Sustained faith that vaccines will be a game-changer, in the face of accumulating evidence that the durability of protection will be fatally short. / / 2. Continued neglect of vectors. Of the (estimated) 4.4 million deaths prevented since 2000, more than 80%, perhaps more than 90%, are attributable to LLINs. In GMAP1, it was estimated that about 70% of total malaria control expenditure would be spent on vector control, and in practice the actual figure was close to 60%. However, according to the plans of GMAP1, less 20% of the research budget was to be spent on vector control. / / Now we face an unprecedented crisis of insecticide resistance, that could sink the whole ship, and we still spend far money on dealing with drug resistance than we do on insecticide resistance. / / And here we are again, preparing GMAP2, and in the next fifteen years it seems we still expect vector control to be responsible for the great majority of deaths prevented, we again expect vector control to occupy about 60% of the total budget, and yet again we intend to spend about 90% of the research on other stuff, and only 10% on vector control. / / Bonkers. / / But when the vector control experts complain, the clinicians and parasitologists laugh gently at our evident bias, as though the entomologists are necessary specialists but unable to see the bigger picture. The alternative is to say "this is a war against transmission, and thus mostly (but not entirely) a war against mosquitoes. Therefore it is the epidemiologists and entomologists who should be the generals who determine overall war strategy. Instead, the generals are all drawn from the medical corps.... / / I'm not saying that's a balanced view, I'm just saying it's about as unbalanced as the situation we have now. / / 3. Continued faith that useful practical applied research can be done by researchers who never talk to programme managers. / / “ |
| “The slow pace in developing new insecticides and drugs” |
| “The unprecedented rise in the investment in malaria research in malaria endemic countries largely due to support from external funders / / Despite this global effort how relatively little domestic funding government in malaria endemic countries continue to invest in malaria research / / The promise of the possibility that a working vaccine may be closer than we had imagined but also the feeble effort that goes into mitigating resistance to existing drugs and insecticides / / Perhaps most surprising to me is that despite the unprecedented investment in malaria research and control very little is actually known empirically about the burden of disease (infection, illness and death) and the world continues to rely on modelled estimates or verbal autopsies. Little has changed in terms of health information systems (routine or surveillance systems) and even less in vital and civil registration systems. / / “ |
| “Lack of an effective vaccine in the market” |
| “Despite the exciting outputs from primary research and the critical need for new tools there still remains a vast gap in research uptake. There is too big a disconnect between researchers and implementers and insufficient people with the skill sets to bridge this gap. And many funders sit firmly at one or other end of the spectrum with very few supporting operational or implementation research.” |
| “The interest attracted from a base of near zero interest in the early 1990s to the international mult-sector interest now” |
| “Failure to invest more in operational research to make better use of existing tools.” |
| “The important, and significant, investment in African Scientists and African science institutions” |
| “How already existing tools applied in an integrated way and tailored to a setting can have great impact with regards to reducing mortality, morbidity and transmission” |
| “The little investments in health systems research - understanding how health systems could improve effective coverage of malaria interventions” |
| “The slow progress from research into policy following the discovery of ACTs while there was evidence that CQ resistance was associated with increased mortality” |
| “Relative limited insight in transcriptomic patterns in plasmodium falciparum.” |
| “The enormous disparity between research and policy, and the parallel universes of the research and international health /donor communities” |

**Responses from National Government**

| “They have outpaced developments in anti-bacterial research / “ |
| --- |
| “The capacity to transmit malaria of assymptomatics PCR positive patients, the whole range of resistance information |
| “The rapid uptake of malaria research findings into policy //2. Malaria vaccine development which has finally seen successful completion // “ |
| “low interest and lack of funding in malaria compared to new diseases such us HIV / few innovations in new effective malaria control tools / |
| “Working together with development partners, funders and other related stakeholders with a common goal in morbidity and mortality reduction. We found that this is possible and we need now to think on the way forward for the next 20 years” |
| “rapid malaria test” |

**Responses from NGOs**

| “The massive mobilization of new resources for such research” |
| --- |
| “Resilience of malaria parasites.” |
| “Lack of a really good vaccine” |
| “Most surprising has been that lack of "true innovation" in malaria community. Our current best tools are still also our oldest. Examples: Bednets are thousands of years old. Artemisinin is 2000 years old and Quinine is 350 years old” |
| “Impressive magnitude in reduction of malaria-related deaths / Increase in political commitment / Alignment around key goals / metrics under the MDG umbrella / Effectiveness of the PDP model in engaging industry to undertake R&D and produce products at an affordable price / The breadth and innovation of the malaria drug pipeline / The speed of emergence of resistance to new interventions” |
| “Difficulty in the development of a vaccine against malaria” |

**Responses classified as other professional affiliation/ or affiliation is missing**

| “How fast the drugs and insecticides can developed and spreading through-out the parasite and vector populations respectively.” |
| --- |
| “The failure to develop a highly effective vaccine.” |
| “The limited progress made in developing a truly effective malaria vaccine” |
| “how long it takes research to feed into implementation” |
