# Supplementary material for: Global priorities for research and the relative importance of different research outcomes: an international Delphi survey of malaria research experts
Source: Malar J. 2016 Dec 6;15:585. doi: 10.1186/s12936-016-1628-4 (PMC5139033; doi:10.1186/s12936-016-1628-4)
Supplement: Supplementary file 3 — Additional file 3. Mean research outcome weights by background. [file 12936_2016_1628_MOESM3_ESM.docx]

**Additional File 3 – Mean research outcome weights by professional background, region and years of experience**

**S1: Mean research outcome weights by professional background**

|  | **Academic (n=35)** | | **National govt (n=7)** | | **International organisation (n=1)** | | **NGO (n=7)** | | **Other (n=4)** | |
| --- | --- | --- | --- | --- | --- | --- | --- | --- | --- | --- |
| **Round 1** | **mean** | **st. err.** | **mean** | **st. err.** | **mean** | **st. err.** | **mean** | **st. err.** | **mean** | **st. err.** |
| Contribution to knowledge | 20.57 | 2.76 | 16.43 | 4.59 | 10.00 | . | 25.71 | 12.51 | 8.75 | 1.25 |
| Benefits to future research and research use | 17.71 | 1.30 | 19.29 | 3.52 | 25.00 | . | 12.14 | 2.86 | 17.50 | 1.44 |
| Benefits from informing policy and product development | 24.86 | 1.75 | 32.86 | 5.44 | 25.00 | . | 19.29 | 4.14 | 27.50 | 7.50 |
| Health and Health sector benefits | 25.71 | 1.78 | 22.86 | 3.25 | 30.00 | . | 23.57 | 4.46 | 32.50 | 7.50 |
| Broader economic benefits | 11.14 | 1.18 | 8.57 | 1.80 | 10.00 | . | 19.29 | 4.14 | 13.75 | 2.39 |
|  |  |  |  |  |  |  |  |  |  |  |
|  | **Academic (n=29)** | | **National govt (n=7)** | | **International organisation (n=1)** | | **NGO (n=6)** | | **Other (n=4)** | |
| **Round 2** | **mean** | **st. err.** | **mean** | **st. err.** | **mean** | **st. err.** | **mean** | **st. err.** | **mean** | **st. err.** |
| Contribution to knowledge | 19.66 | 0.49 | 18.57 | 0.92 | 20.00 | . | 16.67 | 2.47 | 16.25 | 3.75 |
| Benefits to future research and research use | 17.28 | 0.44 | 15.29 | 1.13 | 17.00 | . | 16.00 | 1.37 | 14.00 | 3.00 |
| Benefits from informing policy and product development | 25.10 | 0.33 | 25.71 | 0.71 | 25.00 | . | 25.83 | 0.83 | 26.25 | 1.25 |
| Health and Health sector benefits | 25.55 | 0.29 | 27.43 | 1.27 | 26.00 | . | 26.33 | 0.76 | 29.50 | 3.50 |
| Broader economic benefits | 12.41 | 0.40 | 13.00 | 0.65 | 12.00 | . | 15.17 | 2.43 | 14.00 | 2.00 |

*Respondents where profession is missing are not shown (n=2)

**S2: Mean research outcome weights by region**

|  | **High income (n=31)** | | **Middle income (n=8)** | | **Low income (n=13)** | |
| --- | --- | --- | --- | --- | --- | --- |
| **Round 1** | **mean** | **st. err.** | **mean** | **st. err.** | **mean** | **st. err.** |
| Contribution to knowledge | 16.45 | 1.51 | 21.25 | 4.09 | 13.85 | 2.20 |
| Benefits to future research and research use | 18.55 | 1.07 | 21.25 | 3.37 | 14.62 | 2.00 |
| Benefits from informing policy and product development | 27.26 | 1.76 | 25.00 | 2.99 | 25.00 | 3.71 |
| Health and Health sector benefits | 26.45 | 1.63 | 21.88 | 2.30 | 30.00 | 3.15 |
| Broader economic benefits | 11.29 | 1.23 | 10.63 | 1.99 | 16.54 | 2.36 |
| * Respondents where residence is missing are not shown (n=2) |  |  |  |  |  |  |
|  |  |  |  |  |  |  |
|  | **High income (n=27)** | | **Middle income (n=8)** | | **Low income (n=11)** | |
| **Round 2** | **mean** | **st. err.** | **mean** | **st. err.** | **mean** | **st. err.** |
| Contribution to knowledge | 19.07 | 0.76 | 19.38 | 0.63 | 17.73 | 1.41 |
| Benefits to future research and research use | 16.67 | 0.57 | 16.38 | 0.63 | 16.27 | 1.27 |
| Benefits from informing policy and product development | 25.30 | 0.40 | 25.63 | 0.63 | 25.45 | 0.45 |
| Health and Health sector benefits | 26.00 | 0.49 | 26.13 | 0.13 | 27.09 | 1.30 |
| Broader economic benefits | 12.96 | 0.65 | 12.50 | 0.50 | 13.45 | 0.98 |

**S3: Mean research outcome by years of experience**

|  | **Less than 10 years (n=4)** | | **Between 10 and 20 years (n=15)** | | **More than 20 years (n=33)** | |
| --- | --- | --- | --- | --- | --- | --- |
| **Round 1** | **mean** | **st. err.** | **mean** | **st. err.** | **mean** | **st. err.** |
| Contribution to knowledge | 17.50 | 4.79 | 16.67 | 1.93 | 16.36 | 1.69 |
| Benefits to future research and research use | 16.25 | 2.39 | 19.00 | 2.02 | 17.73 | 1.23 |
| Benefits from informing policy and product development | 33.75 | 2.39 | 27.00 | 3.71 | 25.15 | 1.50 |
| Health and Health sector benefits | 22.50 | 4.33 | 26.00 | 2.14 | 27.42 | 1.78 |
| Broader economic benefits | 10.0 | 0.0 | 11.33 | 2.41 | 13.33 | 1.19 |
| * Respondents where years of experience is missing are not shown (n=2) |  |  |  |  |  |  |
|  |  |  |  |  |  |  |
|  | **Less than 10 years (n=4)** | | **Between 10 and 20 years (n=14)** | | **More than 20 years (n=28)** | |
| **Round 2** | **mean** | **st. err.** | **mean** | **st. err.** | **mean** | **st. err.** |
| Contribution to knowledge | 20.0 | 0 | 19.64 | 0.63 | 18.21 | 0.86 |
| Benefits to future research and research use | 17.0 | 0 | 16.43 | 0.84 | 16.50 | 0.63 |
| Benefits from informing policy and product development | 25.0 | 0 | 25.00 | 0.00 | 25.64 | 0.45 |
| Health and Health sector benefits | 26.0 | 0 | 26.14 | 0.80 | 26.39 | 0.57 |
| Broader economic benefits | 12.0 | 0 | 12.79 | 0.59 | 13.25 | 0.68 |
| * Respondents where years of experience is missing are not shown (n=3) |  |  |  |  |  |  |
|  |  |  |  |  |  |  |
